# Levosimendan and Dobutamin Attenuate LPS-Induced Inflammation in Microglia by Inhibiting the NF-κB Pathway and NLRP3 Inflammasome Activation via Nrf2/HO-1 Signalling

**DOI:** 10.3390/biomedicines12051009

**Published:** 2024-05-03

**Authors:** Federica Mannino, Valentina Urzì Brancati, Rita Lauro, Igor Pirrotta, Michelangelo Rottura, Natasha Irrera, Gian Maria Cavallini, Giovanni Pallio, Eloisa Gitto, Sara Manti

**Affiliations:** 1 Department of Clinical and Experimental Medicine, University of Messina, 98125 Messina, Italy; fmannino@unime.it (F.M.); valeurzi@hotmail.it (V.U.B.); rita.lauro@studenti.unime.it (R.L.); igor.pirrotta@unime.it (I.P.); mrottura@unime.it (M.R.); nirrera@unime.it (N.I.); eloisa.gitto@unime.it (E.G.); 2 Department of Surgery, Medicine, Dentistry and Morphological Sciences, with Interest in Transplants, Oncology and Regenerative Medicine, University of Modena and Reggio Emilia, 41121 Modena, Italy; gianmaria.cavallini@unimore.it; 3 Department of Biomedical and Dental Sciences and Morphological and Functional Imaging, University of Messina, 98125 Messina, Italy; 4 Department of Human Pathology of Adult and Childhood Gaetano Barresi, University of Messina, 98125 Messina, Italy; sara.manti@unime.it

**Keywords:** dobutamine, hypovolemic shock, levosimendan, NFκB, neuroinflammation, NLRP3, Nrf2/HO-1, ROS, Nrf2/HO-1, NFκB

## Abstract

Hypovolemic shock is a circulatory failure, due to a loss in the effective circulating blood volume, that causes tissue hypoperfusion and hypoxia. This condition stimulates reactive oxygen species (ROS) and pro-inflammatory cytokine production in different organs and also in the central nervous system (CNS). Levosimendan, a cardioprotective inodilator, and dobutamine, a β1-adrenergic agonist, are commonly used for the treatment of hypovolemic shock, thanks to their anti-inflammatory and antioxidant effects. For this reason, we aimed at investigating levosimendan and dobutamine’s neuroprotective effects in an “in vitro” model of lipopolysaccharide (LPS)-induced neuroinflammation. Human microglial cells (HMC3) were challenged with LPS (0.1 µg/mL) to induce an inflammatory phenotype and then treated with levosimendan (10 µM) or dobutamine (50 µM) for 24 h. Levosimendan and dobutamine significantly reduced the ROS levels and markedly increased Nrf2 and HO-1 protein expression in LPS-challenged cells. Levosimendan and dobutamine also decreased p-NF-κB expression and turned off the NLRP3 inflammasome together with its downstream signals, caspase-1 and IL-1β. Moreover, a reduction in TNF-α and IL-6 expression and an increase in IL-10 levels in LPS-stimulated HMC3 cells was observed following treatment. In conclusion, levosimendan and dobutamine attenuated LPS-induced neuroinflammation through NF-κB pathway inhibition and NLRP3 inflammasome activation via Nrf2/HO-1 signalling, suggesting that these drugs could represent a promising therapeutic approach for the treatment of neuroinflammation consequent to hypovolemic shock.

## 1. Introduction

Shock is defined as a decreased systemic perfusion and inadequate blood supply to peripheral tissues, resulting in the imbalance between required and provided oxygen during metabolic processes. Basing on its aetiology, shock can be classified as cardiogenic, restrictive (or distributive), and hypovolemic; in particular, cardiogenic shock is due to primary cardiac dysfunction and insufficient cardiac output (CO) [1]. An increased arteriolar vasodilatation with a reduction in systemic vascular resistance (SVR) is one of the features of restrictive or distributive shock, which leads to hypotension and inadequate peripheral perfusion, with or without concomitant decreased CO [2]. Anaphylaxis, neurogenic shock, and septic shock are the most common causes of distributive shock [3]; hypovolemic shock is associated with an important loss of intravascular volume with a reduction in preload, stroke volume (SV), and CO. Different clinical conditions result in hypovolemic shock, including massive haemorrhage, sustained fluid loss without replacement during diarrhoea, vomiting, or heat stroke, and fluid moving from vascular to non-vascular body compartments due to intestinal obstruction, cirrhosis, or burn injuries [3].

The shock incidence is 0.3–0.7/1000 people per year and haemorrhagic shock represents one of the most common causes of admission to Intensive Care Units, while hypovolemic shock mostly occurs in children due to diarrheal illness, especially in developing countries [4]. 

During shock, hypoperfusion causes cellular stress with an increase in reactive oxygen species (ROS) production, with consequent inflammation that compromises the function of vital organs like the heart, liver, kidneys and brain. A prolonged hypoperfusion in the central nervous system (CNS) leads to the lack of essential components of cerebral metabolism, such as oxygen and glucose, decreased ATP production, variations of Ca^2+^ homeostasis, augmented lactate production with an alteration in pH, and inflammation [5]. Moreover, the alteration of the blood–brain barrier (BBB) consequent to cerebral hypoperfusion may induce neurotoxin and pro-inflammatory cytokine crossing in the nervous tissues, thus causing direct damage to the neuronal cells [6].

Together with ROS, also other molecules could be released by damaged cells during shock, such as so-called damage-associated molecular patterns (DAMPs), including heat shock proteins, uric acid, and fragments of the extracellular matrix. DAMPs bind to pattern recognition receptors (PRRs) and trigger the nuclear factor kappa-light-chain-enhancer of activated B cells (NF-κB), signalling and, consequently, activating the Nod-like receptor protein 3 (NLRP3) inflammasome [7]. NLRP3 activation leads to the enzymatic cleavage of pro-caspase 1 into caspase-1, and this enzyme, in turn, activates the pro-inflammatory cytokines interleukin (IL)-1β and IL-18 [8]. Furthermore, NF-κB activates other pro-inflammatory mediators, such as IL-6 and Tumor Necrosis Factor alpha (TNF-α), amplifying the inflammatory response [9]. 

Therefore, the primary goal of managing any shock condition is based on the restoration of vital organs’ perfusion, which can be achieved via resuscitative agents able to replenish intravascular volume, like crystalloid or colloid fluids and vasopressor drugs. In particular, vasopressor drugs include endogenous catecholamines, such as epinephrine, norepinephrine, and dopamine, and exogenous catecholamines like isoproterenol, phenylephrine, milrinone, and dobutamine. Among these, dobutamine, a β1 and α1 receptor agonist, has been used as a first-line inotropic agent to enhance cardiac contractility [10,11]. Dobutamine exerts its beneficial effects through CO and arterial blood pressure increase, thus raising the cerebral blood flow in ischemic brain tissue in patients after hemorrhagic or septic shock even if it does not cross the BBB [12]. Moreover, an in vitro study reported that the antioxidant effects of dobutamine increase heme-oxygenase-1(HO-1) production and the nuclear translocation of nuclear factor erythroid 2-related factor 2 (Nrf2) [13], which have a fundamental role in maintaining redox homeostasis and balance [14]. In addition to inotropic agents, calcium sensitizers have also been used for shock treatment. In this regard, levosimendan acts by increasing the calcium sensitivity of troponin C in cardiomyocytes, thus increasing heart contractility; in addition, this drug is able to cross the BBB with positive effects in the CNS in terms of arterial vasodilation and neuroprotection [15]. In particular, previous papers showed that levosimendan can inhibit phosphodiesterase III (PDE III), preventing cAMP degradation [16] and inducing vasodilation by opening ATP-dependent potassium channels as well as large conductance calcium-activated potassium channels [17,18]. In addition to these inotropic effects, levosimendan might exert anti-inflammatory activity by inhibiting ROS release in polymorphonuclear leukocytes [19] and reducing IL-6 and IL-8 expression in endothelial cells [20,21,22]; moreover, levosimendan is able to inhibit NF-κB activation and reduce TNF-α levels [22,23]. Although previous studies demonstrated that levosimendan and dobutamine have benefits in cardiovascular diseases, the antioxidant and anti-inflammatory effects of these drugs in the CNS have not been deeply explored. Therefore, the aim of this study was to investigate the neuroprotective effects of levosimendan and dobutamine, two drugs commonly used in clinical practice to treat shock, in an “in vitro” model of LPS-induced neuroinflammation.

## 2. Materials and Methods

### 2.1. Cell Culture 

Human Microglial cells (HMC3) were purchased from ATCC (ATCC, Manassas, VA, USA). The HMC3 cells were cultured in Eagle’s Minimum Essential Medium (EMEM), (Sigma-Aldrich, Milan, Italy), supplemented with 1% antibiotic (penicillin/streptomycin), (Sigma-Aldrich, Milan, Italy) and 10% fetal bovine serum (FBS), (ATCC, Manassas, VA, USA) and then incubated at 37 °C with 5% CO_2_. The culture’s medium was replaced every 2 days and the cells were then re-plated.

### 2.2. Cell Treatments

The HMC3 cells were seeded in six-well culture plates at a density of 1.5 × 10^6^ cells/well and incubated at 37 °C with 5% CO_2_ overnight. The day after, cells were challenged with LPS (Escherichia coli serotype 055:B5; Sigma-Aldrich, Milan, Italy) at the dose of 0.1 μg/mL alone or with levosimendan 10 μM (Orion Pharma, Milan, Italy) or dobutamine 50 μM (Hikma Pharmaceuticals, London, UK), for 24 h. Levosimendan and dobutamine concentrations were titrated on the basis of their effects on IL-1β expression (Supplemental Figure S1) while the induction of the neuro-inflammatory model was in accordance with previous published papers [24,25].

### 2.3. MTT Assay

Cell viability was evaluated by an MTT assay. The HMC3 cells were plated in 96-well plates at a density of 1 × 10^5^ cells/well; the day after, cells were incubated with LPS, LPS + levosimendan, or LPS + dobutamine for 24 h. Five hours before the end of the treatment period, 20 µL of tetrazolium dye MTT 3-(4,5-dimethylthiazol-2-yl)-2,5-diphenyltetrazolium bromide (Alfa Aesar, Heysham, UK), dissolved in sterile phosphate buffered saline (PBS), was added into each well. After 24 h of treatment, 200 μL/well of dimethyl sulfoxide (DMSO) (Sigma Aldrich, Milan, Italy) was added to dissolve the insoluble formazan crystals. Cell viability was measured by using a VICTOR Multilabel Plate Reader (Perkin Elmer; Waltham, MA, USA) at λ 540 and 620 nm. Results were expressed as the percentage of cell viability compared to untreated cells [26].

### 2.4. Intracellular ROS Production

Intracellular ROS production was evaluated by using a 5-(and-6)-chloromethyl-2′,7′-dichlorodihydrofluorescein diacetate (CM-H2DCFDA) probe (Thermo Fisher, Milan, Italy) in HMC3 cells challenged with LPS alone or with levosimendan or dobutamine for 24 h. At the end of the treatment, cells were incubated with a CM-H2DCFDA probe (5 μM) for 1 h at 37 °C with 5% CO_2_ and then washed with sterile PBS 2–3 times. After washing, cells were observed with a fluorescent microscope [27]. Fluorescence quantification was performed by using ImageJ software for Windows (Softonic, Barcelona, Spain).

### 2.5. Immunofluorescence 

The HMC3 cells were seeded in 8-well chamber slides at a density of 2.5 × 10^4^ cells/well and challenged with LPS alone or with levosimendan or dobutamine for 24 h. Cells were then fixed with 4% paraformaldehyde (PFA) in 0.2 M phosphate buffer (pH 7.4) for 10 min at 37 °C with 5% CO_2_; after three washes with sterile PBS, the HMC3 cells were pre-incubated with 0.3% triton X-100 for 10 min at RT in order to permeabilize the membranes and with 1% bovine serum albumin (BSA) for 1 h at RT in order to block nonspecific binding sites. Later, the cells were incubated with primary antibodies for Nrf-2, p-NF-κB, and HO-1 (Cell Signaling, Danvers, MA, USA) overnight at 4 °C. The day after, cells were washed three times with PBS and incubated with a FITC-conjugated IgG anti-rabbit antibody (GeneTex, Irvine, CA, USA) diluted in 500 μL of 0.1% BSA for 1 h at RT protected from light. Nuclei were stained with DAPI (Thermo Fisher, Milan, Italy) 1:1000 diluted in PBS for 10 min at RT. Finally, cells were washed three times with PBS and the coverslips were mounted on slides. Immunofluorescence reactions were observed and images were acquired with a fluorescent microscope (Olympus, Milan, Italy); figure montages were prepared by using Adobe Photoshop 7.0 (Adobe System, Palo Alto, CA, USA) [28]. Fluorescence quantification was performed by using ImageJ software for Windows (Softonic, Barcelona, Spain).

### 2.6. Real-Time Quantitative PCR (RTqPCR)

Total RNA was isolated from the HMC3 cells with a Trizol LS Reagent Kit (Life Technologies, Milan, Italy) and then quantified with a NanoDrop Lite spectrophotometer (Thermo Fisher, Milan, Italy). A total of 1 μg of total RNA was reverse-transcribed in a final volume of 20 μL by using the Superscript IV RT Master Mix (Invitrogen, Carlsbad, CA, USA). A total of 1 μL of cDNA was added to the BrightGreen qPCR Master Mix (ABM, Richmond, BC, Canada) together with specific primers at the concentration of 10 μM in a total volume of 20 μL/well to evaluate IL-1β, IL-6, Il-10, NLRP3, Caspase-1, and TNF-α mRNA expression. A qPCR reaction was monitored by using the QuantStudio 6 Flex Real-Time PCR System (Thermo Fisher, Milan, Italy); GAPDH was used as housekeeping gene and the amplified PCR products were quantified by measuring the cycle thresholds (CT) of the target genes and GAPDH. After normalization, the mean value of the control group was chosen as the calibrator and the results were expressed according to the 2^−ΔΔCt^ method, as a fold change relative to the calibrator [29,30,31,32]. Primers used for targets and reference genes are listed in Table 1.

### 2.7. Western Blot 

The HMC3 cells were scraped by using cold RIPA buffer plus proteinase inhibitors and then centrifuged at 15,000 rpm for 15 min at 4 °C. The total protein content was measured in the supernatant with the Bio-Rad protein assay kit (BioRad, Hercules, CA, USA). Proteins (30 μg) were electrophoretically separated on a 10% sodium dodecyl sulphate (SDS) polyacrylamide gel and transferred onto PVDF membranes (BioRad, Hercules, CA, USA) by using a specific Transfer Buffer at 100 V for 1 h. The obtained membranes were blocked with 5% non-fat dry milk for 1 h, washed 3 times in TBS/0.1% Tween buffer, and then incubated with specific primary antibodies for NLRP3 and Caspase-1 (Cell Signaling, Danvers, MA, USA) diluted in TBS/0.1% Tween buffer overnight at 4 °C. The day after, the membranes were washed three times with TBS-0.1% Tween buffer and incubated with a secondary peroxidase-conjugated goat anti-rabbit antibody (KPL, Gaithersburg, MD, USA) for 1 h at RT. Following rinsing with TBS-0.15% Tween buffer, membranes were incubated 1–2 min with the enhanced chemiluminescence system (LumiGlo reserve; Seracare, Milford, MA, USA). Images were obtained and quantified by scanning densitometry by using a bio-image analysis system (C-DiGit, Li-cor, Lincoln, NE, USA). Data were expressed as integrated intensity and β-actin (Cell Signaling, Danvers, MA, USA) was the control for equal loading samples [33,34].

### 2.8. Measurements of Cytokines by Enzyme-Linked Immunosorbent Assay (ELISA)

IL-1β, IL-6, IL-10, and TNF-α protein levels were evaluated in cell supernatants by using an Enzyme-Linked Immunosorbent Assay (ELISA) kits (Abcam, Cambridge, UK), in agreement with the instructions reported by the manufacturer. All samples were assessed in duplicate and the results were interpolated with the standard curves [35].

### 2.9. Statistical Analysis

All the results were expressed as mean ± standard deviation (SD) and the reported values were the results of at least three experiments. All assays were performed in duplicate to ensure reproducibility. The differences between the groups were evaluated with a one-way ANOVA and Tukey’s post-test. A *p*-value less than 0.05 was considered significant and graphs were prepared by using GraphPad Prism (Version 8.0 for macOS, San Diego, CA, USA).

## 3. Results

### 3.1. Levosimendan and Dobutamine Do Not Affect Cell Viability

**Figure 1:**
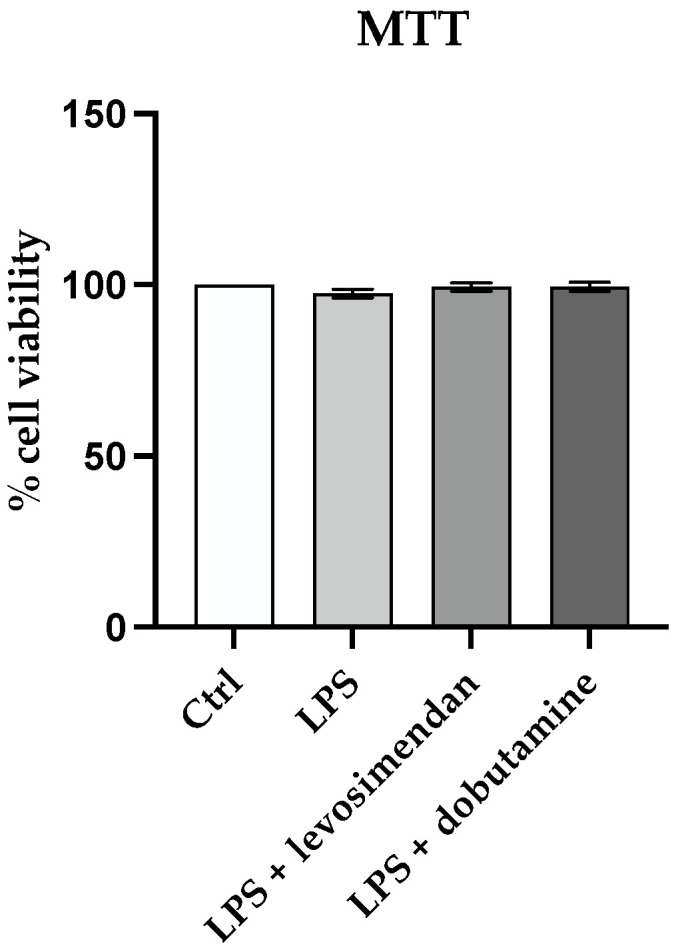
The graph shows the cytotoxicity assay at 24 h in HMC3 cells. The data are expressed as means ± SD of three experiments.

Cell viability was assessed after HMC3 cell incubation with LPS alone or with levosimendan or dobutamine in order to evaluate whether the selected concentrations could have cytotoxic effects. Control cells (unstimulated and untreated) exhibited one hundred percent viability following 24 h. Neither LPS challenge nor the co-incubation with levosimendan or dobutamine affected HMC3 cell viability, thus demonstrating that the used concentrations were appropriate (Figure 1). 

### 3.2. Levosimendan and Dobutamine Reduce Oxidative Stress

**Figure 2:**
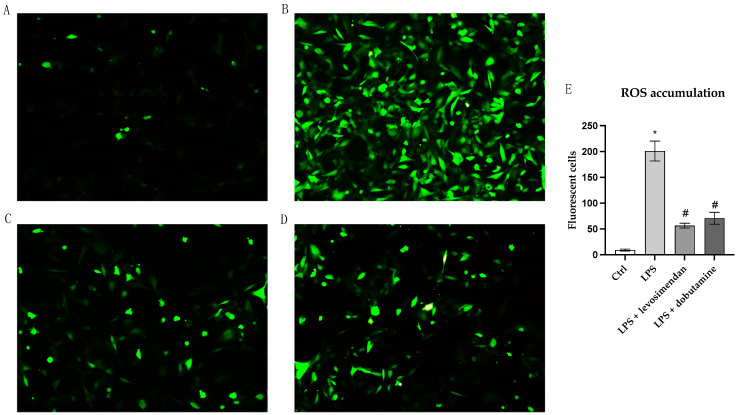
The panel shows intracellular ROS accumulation evaluated by a CM-H2DCFDA fluorescent probe in HMC3 cells from Ctrl (**A**), LPS (**B**), LPS + levosimendan (**C**), and LPS + dobutamine (**D**) groups. Panel (**E**) shows the number of fluorescent cells. All images were captured at 10× magnification. The data are expressed as mean ± SD. * *p* < 0.05 vs. Ctrl; # *p* < 0.05 vs. LPS.

**Figure 3:**
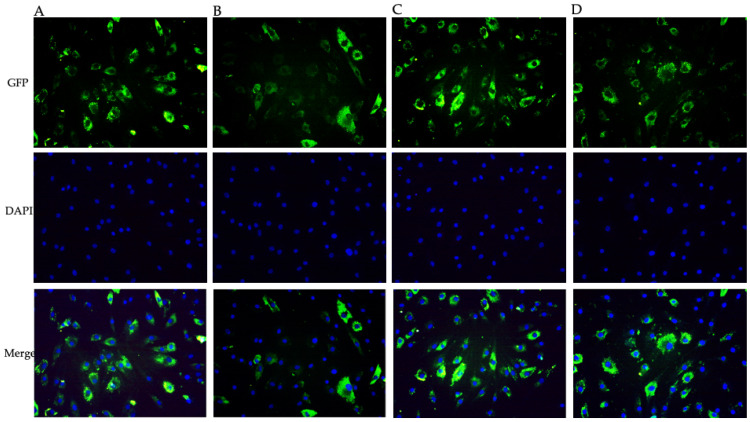
The panel shows immunofluorescence reactions using an anti-Nrf-2 antibody (green fluorescence) in HMC3 cells from Ctrl (**A**), LPS (**B**), LPS + levosimendan (**C**) and LPS + dobutamine (**D**) groups. All images were captured at 10× magnification. GFP: green fluorescent protein; DAPI: 4′,6-diamidino-2-phenylindole.

**Figure 4:**
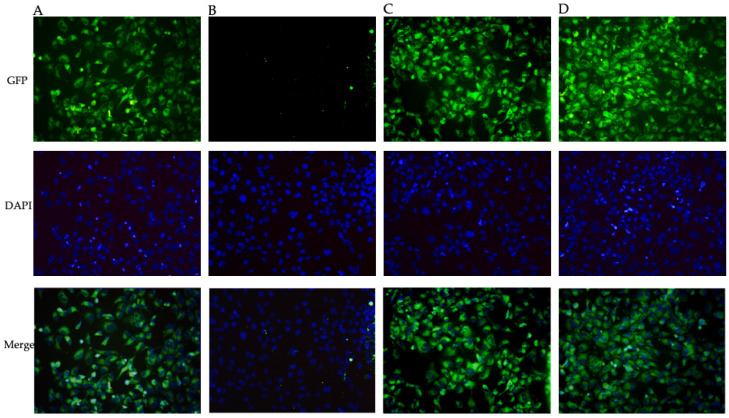
The panel shows immunofluorescence reactions using an anti-HO-1 antibody (green fluorescence) in HMC3 cells from Ctrl (**A**), LPS (**B**), LPS + levosimendan (**C**), and LPS + dobutamine (**D**) groups. All images were captured at 10× magnification. GFP: green fluorescent protein; DAPI: 4′,6-diamidino-2-phenylindole.

**Figure 5:**
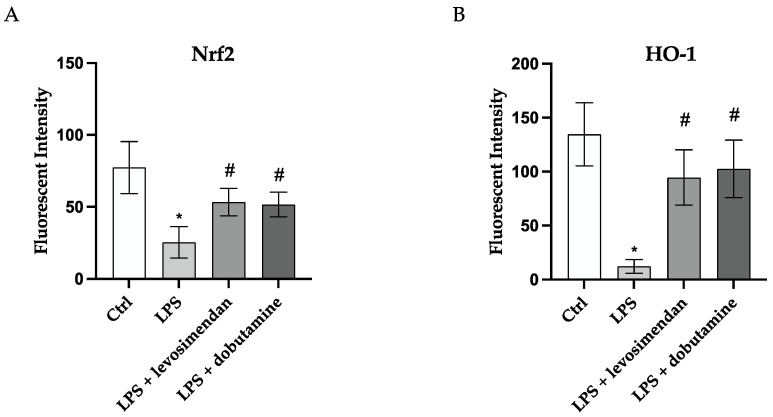
The graphs show Nrf2 (**A**) and HO-1 (**B**) fluorescent quantification performed with Image-J (version 1.54h). The data are expressed as mean ± SD. * *p* < 0.05 vs. Ctrl; # *p* < 0.05 vs. LPS.

Intracellular ROS production was evaluated to explore levosimendan and dobutamine antioxidant effects in this model of neuroinflammation. LPS challenge markedly stimulated intracellular ROS release compared to the control cells (Figure 2B). Instead, intracellular ROS production was significantly reduced in HMC3 cells stimulated with LPS and co-incubated with levosimendan or dobutamine compared to the untreated cells (Figure 2C–E), thus indicating that the use of these drugs might be useful in reducing ROS accumulation during neuroinflammation. To better evaluate the possible antioxidant effects of levosimendan and dobutamine, Nrf2 and HO-1 protein levels, which finely modulate the antioxidant response, were analysed in HMC3 cells. A marked reduction in Nrf2 and HO-1 protein expression was observed in the HMC3 cells stimulated with LPS compared to the controls (Figure 3B and Figure 4B) as a result of the oxidative stress induction triggered by LPS; on the other hand, LPS-challenged cells co-incubated with levosimendan or dobutamine showed a marked increase in Nrf2 and HO-1 protein expression compared to LPS-stimulated cells as demonstrated by the increase in the fluorescence signal showed in Figure 3C,D, Figure 4C,D and Figure 5A,B.

### 3.3. Levosimendan and Dobutamine Reduced Neuroinflammation

**Figure 6:**
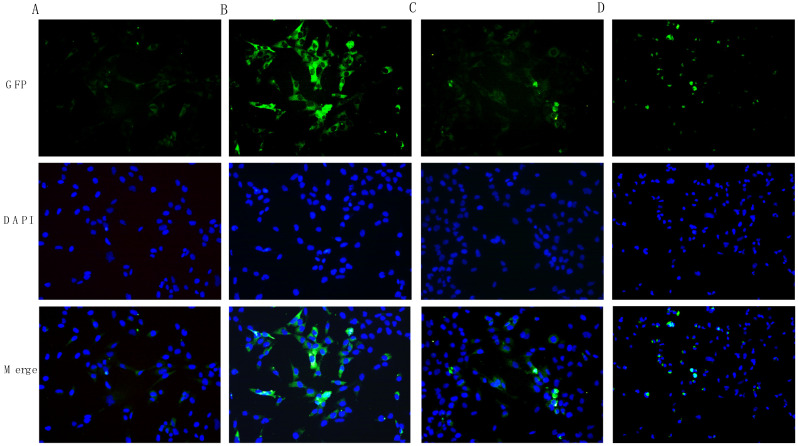
The panel shows immunofluorescence reactions using an anti-p-NF-κB antibody (green fluorescence) in HMC3 cell from Ctrl (**A**), LPS (**B**), LPS + levosimendan (**C**), and LPS + dobutamine (**D**) groups. All images were captured at 10× magnification. GFP: green fluorescent protein; DAPI: 4′,6-diamidino-2-phenylindole.

**Figure 7:**
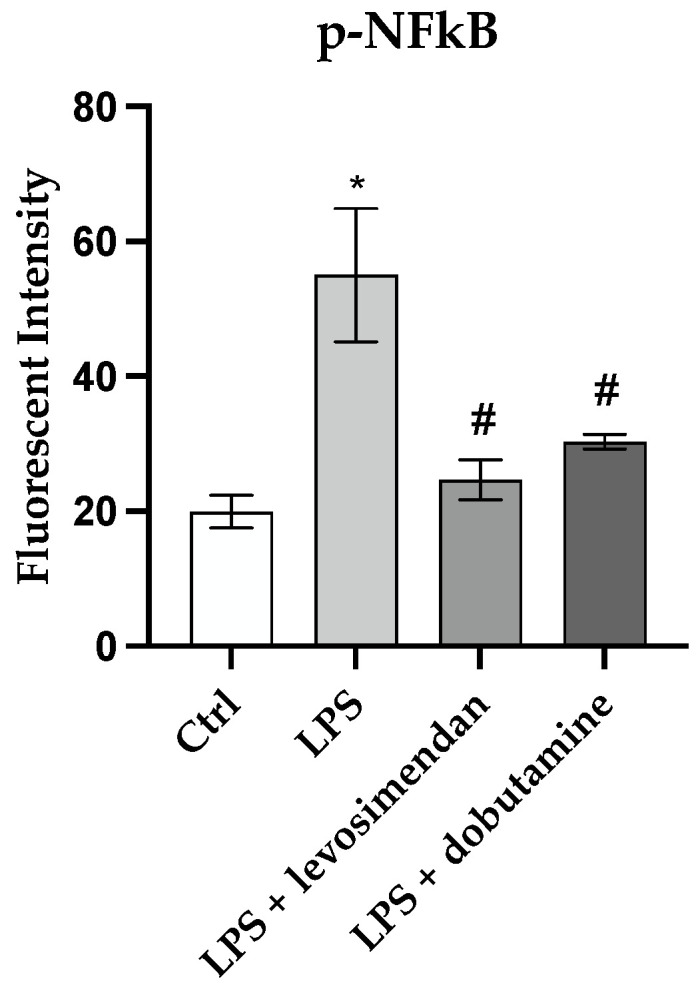
The graph shows p-NF-κB fluorescent quantification performed with Image-J (version 1.54h). The data are expressed as mean ± SD. * *p* < 0.05 vs. Ctrl; # *p* < 0.05 vs. LPS.

**Figure 8:**
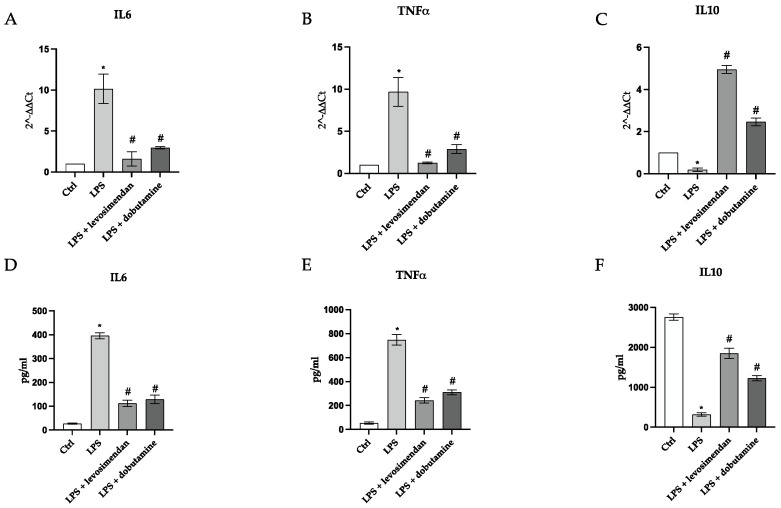
The graphs represent IL-6 (**A**), TNF-α, (**B**), IL-10 (**C**), mRNA expression (RTqPCR analysis) and IL-6 (**D**), TNF-α (**E**), and IL-10 (**F**) protein expression (ELISA assay) in HMC3 cells. The data are expressed as mean ± SD of three experiments. * *p* < 0.05 vs. Ctrl; # *p* < 0.05 vs. LPS.

The gene expression of the transcription factor NF-κB, of the pro-inflammatory mediators TNF-α and IL-6, and the anti-inflammatory cytokine IL-10 were investigated to study levosimendan and dobutamine anti-inflammatory effects in this model of neuroinflammation. The LPS stimulus induced a significant upregulation in p-NF-κB protein expression compared to the control cells (Figure 6B). Both levosimendan and dobutamine treatment significantly reduced p-NF-κB protein expression compared to the LPS-challenged cells (Figure 6C,D and Figure 7). The LPS stimulus and the consequent NF-κB activation also caused a significant increase in TNF-α and IL-6 gene expression as well as a marked decrease in IL-10 mRNA expression compared to the control group (Figure 8A–C). The treatment with levosimendan and dobutamine significantly reduced TNF-α and IL-6 gene expression, and upregulated IL-10 mRNA expression compared to the LPS group (Figure 8A–C). Furthermore, to confirm the anti-inflammatory effects of levosimendan and dobutamine, the mature protein levels were evaluated in the supernatants of the HMC3 cells. LPS-challenged cells showed a marked increase in TNF-α and IL-6 protein expression with a concomitant significative reduction in IL-10 protein levels compared to the control cells (Figure 8D–F). By contrast, levosimendan and dobutamine treatment dampened TNF-α and IL-6 protein levels and increased IL-10 protein expression in the HMC3 cells stimulated with LPS (Figure 8D–F).

### 3.4. Levosimendan and Dobutamine Blunt NLRP3 Signal

**Figure 9:**
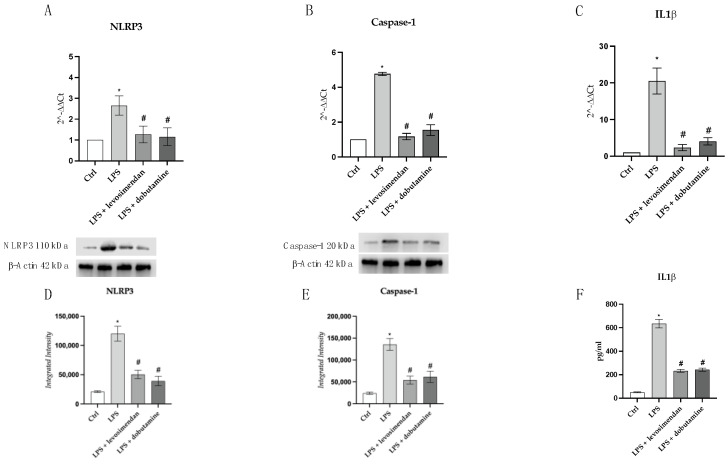
The graphs represent NLRP3 (**A**), Caspase-1 (**B**), IL-1β (**C**), and mRNA expression (RTqPCR analysis) and NLRP3 (**D**), Caspase-1 (**E**), and IL-1β (**F**), protein expression (Western blot analysis and ELISA assay) in HMC3 cells. The targets and the β-actin of panel D and E were detected on the same membrane. The data are expressed as mean ± SD of three experiment. * *p* < 0.05 vs. Ctrl; # *p* < 0.05 vs. LPS.

The effects of levosimendan and dobutamine treatments on the NLRP3 pathway were investigated by using RT-qPCR, Western blotting, and an ELISA assay. LPS stimulation and the consequent NF-κB activation caused a significant increase in the mRNA expression of NLRP3 in HMC3 cells compared to the controls (Figure 9A). Instead, levosimendan or dobutamine treatment caused a marked reduction in NLRP3 mRNA expression compared to LPS-challenged cells (Figure 9A). To deeply explore the inflammatory cascade triggered by NLRP3 activation, we also evaluated its downstream signal and, more specifically, the mRNA expression of caspase-1 and IL-β in the HMC3 cells challenged with LPS. We observed that the LPS stimulus caused a significant increase in caspase-1 and IL-1β mRNA expression compared to the control cells (Figure 9B,C). On the other hand, levosimendan or dobutamine treatment markedly reverted the gene expression increase of the NLRP3 downstream markers in LPS-stimulated cells (Figure 9B,C). To confirm the levosimendan and dobutamine inhibitory effects on the NLRP3 cascade, we also evaluated the mature protein. A significant increase in NLRP3, Caspase-1, and IL-1β protein levels were detected following the LPS challenge compared to those observed in the control cells (Figure 9D–F). On the other hand, levosimendan or dobutamine treatment caused a significant decrease in these protein levels compared to LPS group (Figure 9D–F), indicating that levosimendan and dobutamine can inhibit the NLRP3 pathway during neuroinflammation.

## 4. Discussion

Hypovolemic shock causes tissue hypoperfusion and hypoxia, thus resulting in an increase in ROS production and inflammatory status that affect the function of vital organs like the heart, liver, kidney, and brain [36,37,38,39,40,41]. In particular, the increased oxidative stress in the CNS leads to microglia activation and neuroinflammation. Thus, once activated, microglia trigger pro-inflammatory cytokines and mediators to release, such as IL-1β, IL-6, and TNF-α, leading to neuronal cell degeneration [42,43].

Currently, no specific treatment is available to slow or stop the neuroinflammation consequent to hypovolemic shock; therefore, the use of drugs commonly used in clinical practice to treat shock could be considered a promising therapeutic strategy for treating this condition.

Previous in vitro studies conducted in cardiomyocytes and endothelial cells stimulated with IL-1β or under hypoxic stress, and in polymorphonuclear leucocytes from patients with acute heart failure or septic myocardial depression, showed the antioxidant and anti-inflammatory effects of levosimendan in ROS-related conditions [44,45]. Moreover, dobutamine effectiveness was also demonstrated in ischemic conditions both “in vitro” and “in vivo” through the stimulation of antioxidant genes [46,47]. Oxidative stress may be induced by pathogen-associated molecular patterns (PAMPs), such as LPS, that may activate different molecular pathways, including the NF-κB/NLRP3 pathway, thus playing a role in the pathogenesis of different diseases and also in neuroinflammation, well miming the consequences of hypovolemic shock in the CNS [48,49,50,51].

In this study, we evaluated the neuroprotective effects of levosimendan and dobutamine, two drugs commonly used to treat shock, in an “in vitro” model of LPS-induced neuroinflammation, as well as the mechanistic relationship between their antioxidant activity and the Nrf2/HO-1/NF-κB/NLRP3 axis.

Consistent with previous results supporting the role of ROS in neuroinflammation [52,53,54], our results showed a significant increase in ROS production following LPS challenge in HMC3 cells. Moreover, levosimendan and dobutamine counteracted the LPS-induced ROS increase, confirming data from previous experimental studies [55,56,57].

LPS challenge and the consequent ROS increase significantly reduced Nrf2 and HO-1 expression, which act as key regulators in the protection mechanisms against oxidative stress with a fundamental role in antioxidant metabolism and response, as well as in the modulation of neuroinflammation [58,59,60]. Both levosimendan and dobutamine treatment showed an enhancement of Nrf2 and HO-1 expression compared to LPS-challenged cells; these results were in accordance with previous papers that showed a significant increase in the Nrf2/HO-1 pathway following dobutamine or levosimendan treatment in mice [14,61] and in rats [62]. The up-regulated expression of Nrf2/HO-1 could reduce the inflammatory response through NF-κB signal inhibition [63,64]. Moreover, recent studies revealed that the upregulation of the Nrf2/HO-1 pathway might hinder NLRP3 inflammasome activation both directly and indirectly, through NF-κB inhibition [65,66], also in neuroinflammatory conditions [67,68]. 

The NLRP3 inflammasome complex comprises the pattern recognition receptor NLRP3, the adaptor protein apoptosis-associated speck-like protein containing a CARD (ASC), and the effector caspase-1. This complex is activated in two-steps: the priming signal, referred to as signal 1, originates from microbial components, like LPS, which activate the transcription factor NF-κB, resulting in NLRP3, caspase-1, pro-IL-1β, and pro-IL-18 upregulation. 

The activation signal, known as signal 2, comes from various stimuli, including ROS, which trigger the NLRP3 inflammasome activation that leads to the assembly of its three components and subsequently generates caspase-1 active form. Then, caspase-1 plays a crucial role in producing IL-1β and IL-18 mature cytokines, previously stored as inactive molecules [69]. Previous studies described the role of the NLRP3 inflammasome during neuroinflammation, especially focusing on the TLR4/NF-κB/NLRP3 pathway [70,71]. TLR4 is widely expressed on the surface of microglia and is involved in LPS recognition. After LPS binding to TLR4, microglial cells were activated, thus increasing NF-κB expression and, consequently, NLRP3, caspase-1, and IL-1 β levels [72,73]. In accordance with these previous findings, in our study, the protein expression of p-NF-κB, NLRP3, caspase-1, and IL-1β were upregulated in HMC3 cells stimulated with LPS. In contrast, the treatment with levosimendan or dobutamine significantly downregulated p-NF-κB levels, hindering NLRP3 expression and its downstream signal. Moreover, previous evidence suggested that NF-κB inhibition might be the key to blunt NLRP3 signalling by activating the Nrf2/HO-1 signal pathway [74,75]; therefore, we speculated that the inhibition of oxidative stress and the activation of the Nrf2/HO-1 pathway mediated by levosimendan and dobutamine are involved in NLRP3 inflammasome inhibition during neuroinflammation, although these effects were investigated for the first time in the present study. Moreover, it is well known that LPS activates TLR4/NF-κB signalling, thus stimulating the transcription of the pro-inflammatory cytokines IL-6 and TNFα [76]. In accordance with these previous findings, in our study LPS significantly increased both the mRNA and protein expression of IL-6 and TNFα with a concomitant reduction in the expression of the anti-inflammatory cytokine IL-10. On the other hand, levosimendan and dobutamine treatment significantly reduced both the gene and protein expression of IL-6 and TNFα, by increasing IL-10 levels compared to LPS-challenged cells. These findings are consistent with previous data which demonstrated that levosimendan anti-inflammatory effects were mediated by NF-κB suppression, even if these results were obtained in different experimental models [77,78]. Moreover, dobutamine treatment already showed its anti-inflammatory effects through TNF-α and IL-6 reduction in rats with endotoxemia and in septic shock patients [79,80]; however, in this study, for the first time, we demonstrated that this reduction is associated with NF-κB downregulation. 

## 5. Conclusions

In conclusion, this pioneering study demonstrated that treatment with dobutamine and levosimendan—two drugs commonly used in the clinical practice to treat shock—could alleviate the severity of neuroinflammation related to hypoperfusion, through their antioxidant and anti-inflammatory effects via the activation of the Nrf2/HO-1 pathway and the inhibition of NF-κB/NLRP3 inflammasome signalling. In addition, considering that both levosimendan and dobutamine are currently on the market, they could be easily used for the management of neuroinflammation in patients affected by hypovolemic shock. However, this interesting preclinical evidence deserves to be deeply investigated in a clinical scenario.

## Figures and Tables

**Table 1:** Primer list.

Gene	Sequence
GAPDH	Fw:5′TTTTGCGTCGCCAGCC3′ Rw:5′ATGGAATTTGCCATGGGTGGA3′
IL1-β	Fw:5′AACCTCTTCGAGGCACAAGG3′ Rw:5′AGATTCGTAGCTGGATGCCG3′
TNF-α	Fw:5′GACAAGCCTGTAGCCCATGT3′ Rw:5′GGAGGTTGACCTTGGTCTGG3′
IL-6	Fw:5′CCTTCGGTCCAGTTGCCTTCT3′ Rw:5′TCTGAGGTGCCCATGCTACA3′
IL-10	Fw:5′ACACATCAGGGGCTTGCTC3′ Rw:5′GTGGTCAGGCTTGGAATGGA3′
NLRP3	Fw:5′GCTGGCATCTGGATGAGGAA3′ Rw:5‘GTGTGTCCTGAGCCATGGAA3′
Caspase-1	Fw:5′GAAAAGCCATGGCCGACAAG3′ Rw:5′GCTGTCAGAGGTCTTGTGCT3′

## Data Availability

The data presented in this study are available on request from the corresponding author.

## Supplementary Materials

The following supporting information can be downloaded at: https://www.mdpi.com/article/10.3390/biomedicines12051009/s1, Figure S1: Levosimendan and dobutamine dose response experiment.
