# Supplementary figures and images for: Levosimendan and Dobutamin Attenuate LPS-Induced Inflammation in Microglia by Inhibiting the NF-κB Pathway and NLRP3 Inflammasome Activation via Nrf2/HO-1 Signalling
Source: Biomedicines. 2024 May 3;12(5):1009. doi: 10.3390/biomedicines12051009 (PMC11117907; doi:10.3390/biomedicines12051009)

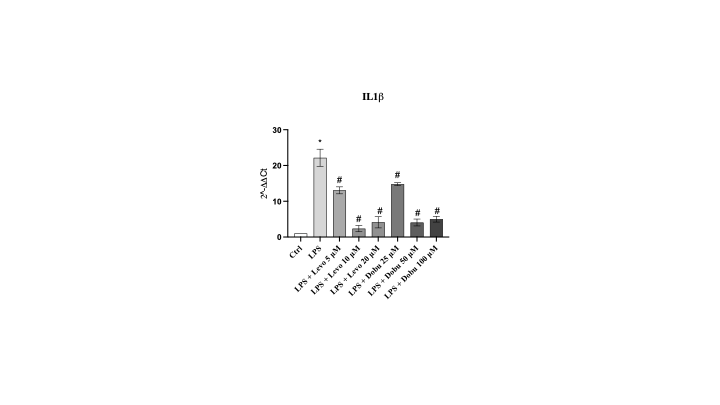

Supplement: Supplementary file 1 [file biomedicines-12-01009-s001.zip › Figure S1.tiff]
